# Patients' Perceptions of Physician-Patient Discussions and Adverse Events with Cancer Therapy

**DOI:** 10.1111/j.1753-5174.2008.00011.x

**Published:** 2008-09

**Authors:** Dawn Hershman, Elizabeth Calhoun, Kinga Zapert, Shawn Wade, Jennifer Malin, Rich Barron

**Affiliations:** * Department of Medicine and the Herbert Irving Comprehensive Cancer Center, College of Physicians and SurgeonsNew York, USA; † University of IllinoisChicago, IL, USA; ‡ Health Care and Public Policy, Harris InteractiveRochester, New York, USA; § Harris InteractiveRochester, New York, USA; ¶ Global Health EconomicsAmgen, Thousand Oaks, CA, USA; ** Global Health EconomicsAmgen, Thousand Oaks, CA, USA

**Keywords:** Chemotherapy, Side-Effects, Communication

## Abstract

**Objectives:**

Patients with cancer who are treated with chemotherapy report adverse events during their treatment, which can affect their quality of life and increase the likelihood that their treatment will not be completed. In this study, patients' perceptions of the physician-patient relationship and communication about cancer-related issues, particularly adverse events were examined.

**Methods:**

We surveyed 508 patients with cancer concerning the occurrence of adverse events and their relationship and communication with their physicians regarding cancer, treatment, and adverse events.

**Results:**

Most individuals surveyed (>90%) discussed diagnosis, treatment plan, goals, and schedule, and potential adverse events with their physicians before initiating chemotherapy; approximately 75% of these individuals understood these topics completely or very well. Physician-patient discussions of adverse events were common, with tiredness, nausea and vomiting, and loss of appetite discussed prior to chemotherapy in over 80% of communications. These events were also the most often experienced (ranging in 95% to 64% of the respondents) along with low white blood cell counts (WBCs), which were experienced in 67% of respondents. Approximately 75% of the individuals reported that their overall quality of life was affected by adverse events.

**Conclusions:**

These findings suggest that discussions alone do not provide patients with sufficient understanding of the events, nor do they appear to adequately equip patients to cope with them. Therefore, efforts to improve cancer care should focus on developing tools to improve patients' understanding of the toxicities of chemotherapy, as well as providing resources to reduce the effects of adverse events.

Most patients who are treated with standard chemotherapy regimens report adverse events—including fatigue, nausea, diarrhea, and pain—during treatment [1,2], but the incidence of chemotherapy-related adverse events and their effect on doctors and nurses have been underestimated. A community study in 12,239 women with breast cancer that was conducted in 2006 found that serious chemotherapy-related adverse events were more common than had been reported in large clinical trials and led to more healthcare expenditures than had been estimated [3].

The occurrence of adverse events increases the likelihood that patients will not complete their treatment [4–7]. Febrile neutropenia, for example, is not directly linked to survival but can lead to life-threatening infections [8,9] and chemotherapy dose reductions and delays, which can compromise survival [10–13]. Furthermore, quality of life may be compromised in patients with cancer who have chemotherapy-induced neutropenia [14], fatigue or anemia [15–17], nausea or vomiting [18,19], or weight loss [20].

Research in physician-patient communication has shown that effective communication and active patient participation in making decisions about treatments can influence a patient's satisfaction, quality of life, and treatment outcome [21–26]. In particular, verbal and nonverbal communication between physicians and patients can affect a patient's satisfaction [27,28], adherence [29], and quality of life [30,31]. Insufficient communication or miscommunication may compromise a patient's ability to participate in making decisions about treatment [21].

This study examines patients' perceptions of the physician-patient relationship and communication about cancer-related issues, particularly adverse events. We also performed an exploratory assessment of differences in race concerning patients' perceptions about physician-patient communication about cancer-related topics and patients' understanding of these topics.

## Methods

### Patients

Individuals surveyed were recruited from December 2003 through January 2004 from the Harris Interactive Chronic Illness Panel and from NexCura and were invited to participate in a secure Internet survey of their opinions on health-related issues. Participants were invited to complete the online survey if they met the following criteria: (i) age ≥18 years; (ii) had been treated for cancer within the past 12 months; and (iii) had a diagnosis of lung, breast, colon, pancreatic, or ovarian cancer, lymphoma (Hodgkin's or non-Hodgkin's), multiple myeloma, or sarcoma.

The Harris Interactive Chronic Illness Panel consists of more than 2 million US persons who have been screened for chronic illnesses from the Harris Interactive general panel of more than 5 million US persons. The cancer subpanel was used as the primary sample source for all survey respondents. NexCura provides a variety of healthcare information and communications services, including online education for patients, and maintains a database of more than 500,000 persons with various illnesses, including cancer.

The participants were given different compensation, according to the source of recruitment. Patients recruited from the Harris Interactive Chronic Illness Panel were compensated with 100 HIpoints, the currency of incentive with which Harris Interactive compensates participants in its surveys. There is no direct conversion between HIpoints and US currency, but 800 HIpoints, for example, can be redeemed for a $5 gift certificate at retail stores. Patients recruited through NexCura were paid $25. The study did not collect protected private health information and was IRB exempt.

### Survey

Participants completed an Internet survey that required approximately 20 minutes to complete. The survey questions were obtained from Harris Poll's question bank and were customized to the cancer experience. They addressed patients' experiences with cancer treatment. Specifically, discussions about treatment and adverse events were posed (e.g., Before you started chemotherapy, did you and your doctor or nurse discuss any of the following: treatment expectations, possibility of delays, potential side effects, etc?). Patient understanding of the information was asked (e.g., How well would you say you understand each of the following: prognosis, treatment expectations, potential side effects, etc, as well as adverse events that had occurred?). Questions concerning patients' perceptions of their communication and relationship with their physician were also asked. For example, the following question was asked: Overall, how would you rate your relationship with your doctor? All survey questions are available on request from the authors.

### Statistical Analysis

Final data obtained from the respondents were weighted to match the characteristics of the targeted cancer patient population by using the following parameters: age, gender, race or ethnicity, education level, geographic region, and household income. Demographic targets were estimated from an internal Harris Interactive national healthcare survey of chronically ill patients, including a large number of patients with cancer.

## Results

### Study Participants

Five hundred eight persons were recruited through an Internet survey to participate in the study. A total of 28,075 persons were solicited (8075 from the Harris Interactive Chronic Illness Panel and 20,000 from NexCura). Of the 28,075 persons, 2807 responded, 540 qualified, and 508 completed the survey. Individuals qualified for this study if they answered in the affirmative to the initial screening questions that included the inclusion criteria. The participants were predominantly white (87%) and married (71%); other demographic characteristics, such as age (65 years or older), education, and income, were more variably distributed (Table 1). Eighty-two percent of respondents had been treated with chemotherapy, and 27% reported that their cancer had recurred after being in remission.

**Table 1:** Patient demographics

	No. (%) of patients (N = 508)
Sex*	
Female	324 (64)
Male	185 (36)
Age, year*	
<35	25 (5)
35–49	92 (18)
50–64	139 (27)
≥65	253 (50)
Ethnicity*	
White	444 (87)
Black	32 (6)
Hispanic	14 (3)
Other	9 (2)
Declined to state	10 (2)
Education	
High school or less	240 (47)
Some college	151 (30)
College graduate	117 (23)
Annual household income, $*	
<35,000	143 (28)
35,000–75,000	113 (22)
>75,000	127 (25)
Declined to state	126 (25)
Household status	
Married or living with partner	359 (71)
Single, never married	29 (6)
Divorced, separated, widowed	121 (24)
Overall health*	
Excellent or very good	104 (20)
Good	188 (37)
Fair or poor	217 (43)
Cancer recurred after remission	136 (27)

* It is typical for the sum of the rounded weighted frequencies to be off by 1 or 2 from the overall base size for the question (N = 508).

### Patient-physician Communication

**Figure 1:**
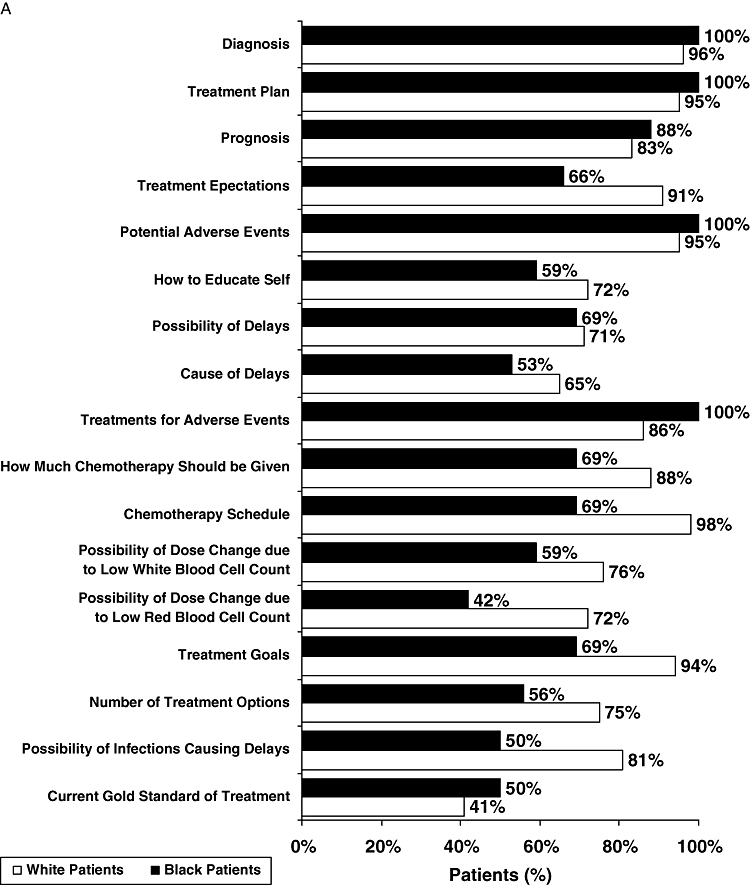

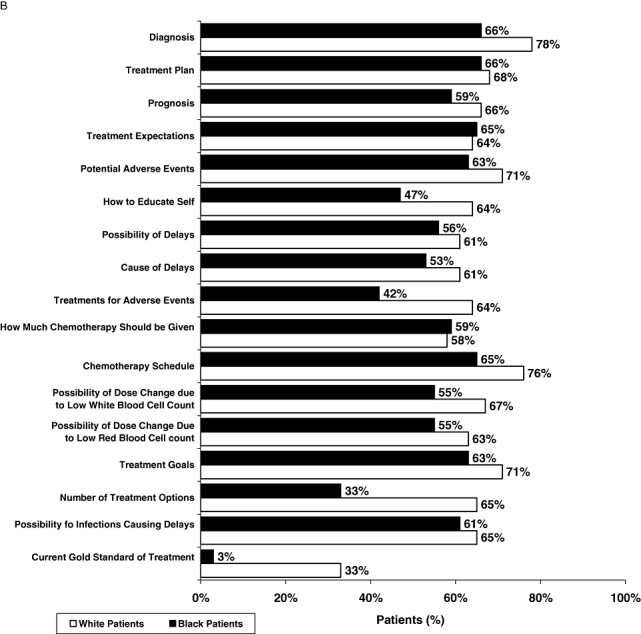
Physician-patient discussions and patient understanding of information on cancer-related topics. (A) Patients who discussed cancer-related topics with their physician. (B) Patients who understood the information provided.

More than 90% of respondents reported that they had had discussions with their physicians about the most fundamental aspects of cancer treatment before starting chemotherapy. The topics discussed included diagnosis, treatment plan, treatment schedule, treatment goals, and adverse events (Table 2). Slightly fewer individuals (80% to 90%) reported having discussed prognosis, expectations of treatment, doses of chemotherapy, and medications for adverse events (see Table 2). At least two thirds of respondents reported having discussed how to educate themselves about cancer and the possibility and causes of treatment delays along with the possibility of treatment delays or reductions due to low WBCs or RBCs. However, the proportion of respondents who described themselves as understanding these topics “completely” or “very well” was generally lower for each topic than the proportion who reported having discussed it (see Table 2). The proportion of black respondents who reported that they had discussed these topics with their physicians and understood them was lower than that of white respondents (Figure 1). Overall, the majority of the individuals reported having had good relationships and good communication with their physicians, with more than 90% of respondents expressing satisfaction and trust. Moreover, many reported that they were comfortable with asking their physicians questions.

**Table 2:** Physician-patient discussions and patient understanding of cancer-related topics

	No. (%) of patients (N = 508)		
Topic	Patients who discussed topic before chemotherapy	Patients who understood topic “completely” or “very well”	Patients who understood topic “somewhat” or “fairly well”
Diagnosis	489 (96)	393 (77)	109 (21)
Treatment plan	485 (95)	343 (68)	157 (31)
Prognosis	428 (84)	341 (67)	131 (26)
Expectations of treatment outcomes	452 (89)	334 (66)	160 (31)
Potential adverse effects of chemotherapy	486 (96)	359 (71)	144 (28)
How to educate oneself about cancer	365 (72)	316 (62)	163 (32)
The possibility of treatment delays	361 (71)	316 (62)	156 (31)
The cause of treatment delays	334 (66)	317 (62)	161 (32)
The medications used to prevent and manage adverse events	443 (87)	326 (64)	156 (31)
How much chemotherapy to be given	445 (88)	305 (60)	157 (31)
The chemotherapy schedule	488 (96)	384 (76)	108 (21)
The possibility of dose delays or reductions because of a low white blood cell count	386 (76)	341 (67)	129 (25)
The possibility of dose delays or reductions because of a low red blood cell count	359 (71)	317 (62)	157 (31)
The goals of treatment	472 (93)	363 (71)	129 (25)
The current “gold standard” of treatment	203 (40)	153 (30)	139 (27)
The number of treatment options available	366 (72)	265 (52)	184 (36)
The possibility that infections can cause treatment delays	408 (80)	330 (65)	163 (32)

### Discussion and Experience of Adverse Events

**Figure2:**
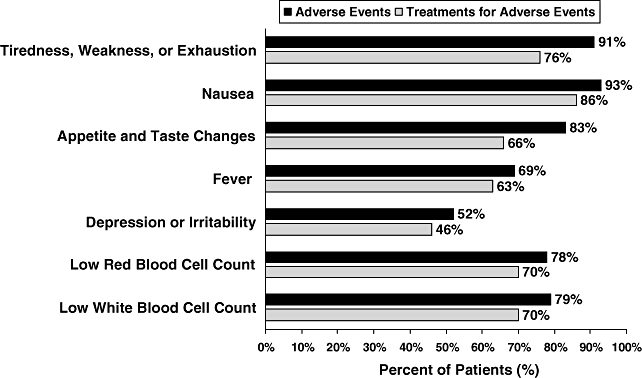
Physician-patient discussions about cancer- or chemotherapy-related adverse events. Patients who recalled a discussion about the adverse event are shown in black and patients who recalled a discussion about supportive care or treatment for the adverse event are shown in gray.

Ninety-six percent of respondents reported that they had discussed adverse events with their physicians before starting chemotherapy (see Table 2), but their responses to a list of common adverse events showed that some adverse events were addressed in 75% or fewer of those discussions (Table 3). Specifically, serious adverse events such as fever and chills (69%) and low white blood cell counts (79%) were discussed less frequently than events such as nausea (93%) and tiredness and weakness (91%). However, it is encouraging to discover that the proportion of individuals discussing adverse events were similar to the proportion of those who also discussed treatment for those adverse events (Figure 2).

**Table 3:** Physician-patient discussions and patient experience of adverse events

	No. (%) of patients (N = 508)		
Adverse event	Patients who discussed symptoms of adverse event before chemotherapy	Patients who discussed treatments for adverse event before chemotherapy	Patients in whom adverse events occurred
Tiredness, weakness, or exhaustion	462 (91)	386 (76)	484 (95)
Nausea, vomiting	470 (93)	437 (86)	323 (64)
Loss of appetite, changes in taste	422 (83)	334 (66)	376 (74)
Fever (temperature, chills)	352 (69)	320 (63)	233 (46)
Depression or irritability	264 (52)	232 (46)	269 (53)
Low white blood cell count (neutropenia)	399 (79)	355 (70)	342 (67)
Low red blood cell count (anemia)	395 (78)	357 (70)	282 (56)

Many respondents also reported the occurrence of adverse events during treatment (see Table 3). The frequencies of such events ranged from 95% for tiredness, weakness, or exhaustion to 46% for fever (temperature, chills). Interestingly, the frequencies of discussions of adverse events paralleled the frequencies of the occurrence of these events: 91% of respondents reported that they had discussed symptoms of fatigue with their physicians, and 95% reported that they had experienced it; 52% reported that they had discussed symptoms of depression, and 53% reported that they had experienced it. This may indicate that patients have selective memories about discussions with their physicians before starting chemotherapy or that the frequency with which physicians discuss adverse events relates to how frequently those events typically occur.

### Effects of Cancer Treatment–related Adverse Events

The majority of respondents with adverse events said that those events had had negative effects on their quality of life (75%), ability to enjoy daily activities (67%), ability to work (66%), ability to carry out everyday activities (60%), and ability to get back to a normal life (56%) (Table 4).

**Table 4:** Effects of adverse events on patients

Effects of adverse event on patient's	No. (%) of patients* (N = 497)†
Quality of life	389 (78)
Enjoyment of daily activities	334 (67)
Ability to work	326 (66)
Ability to carry out everyday activities	297 (60)
Ability to get back to a normal life	278 (56)
Ability to maintain relationships	227 (46)

* It is typical for the sum of the rounded weighted frequencies to be off by 1 or 2 from the overall base size for the question (N = 508).

† Data are from patients in whom at least 1 adverse event occurred.

The respondents’ experiences with neutropenia illustrate the discrepancy between physician-patient communication and patient understanding. For example, 76% of individuals surveyed reported having discussed with their physicians the possibility of chemotherapy modifications because of neutropenia, but only 68% reported understanding this information “completely” or “very well.” Also, more than 90% of respondents reported chemotherapy dose modifications during their treatment, but the possibility of dose delays and modifications was discussed before the start of chemotherapy in only 65% to 70% of cases.

## Discussion

Most respondents reported having a good relationship and extensive discussions with their physicians before beginning chemotherapy about not only basic aspects of their illness and cancer treatments but also the adverse events that might occur. However, serious adverse events such as fever and chills and low white blood cell counts were discussed less frequently than events such as nausea, tiredness, and weakness. Moreover, fewer individuals felt that they fully understood the information they had been given. Also, even though the majority of respondents reported having discussed many cancer-related topics with their physicians, those discussions alone do not necessarily translate into patient understanding. Furthermore, the low frequency of discussions about some of the less-common adverse events suggests a need for improvement in communication, as not all respondents who had adverse events had discussed them with their physicians before starting chemotherapy.

There remains a need for developing and using educational resources for patients with cancer. The disparity between the number of patients who believe that these programs are important for recovery (75%) and the low enrollment rate in them (7%) suggests that much remains to be done to encourage and facilitate patient involvement in these valuable resources and to understand the barriers to patient involvement that exist.

Furthermore, even though the sample size was insufficient for statistical analysis of the responses by race, this exploratory assessment suggests that black patients do not discuss with their physicians and therefore do not fully understand detailed topics related to chemotherapy and possibilities of dose delays and schedules as often as white patients. Therefore, these factors may lead to a decrease in quality of life and ultimately survival. These observations are consistent with the findings in previous research reporting that 5-year survival rates are lower in black patients than in white patients in all major cancer types [32–36]. Also, numerous factors have been explored to explain the lower survival in black patients with cancer, but the influence of cancer treatment−related adverse events has not been well explored. Due to these exploratory assessments, we believe these issues merit further evaluation.

The results of this study should be interpreted in light of several limitations. For example, participants were recruited from two cohorts of subjects who are enrolled in Internet-based survey programs. Our results were weighted to reflect the demographic characteristics of a general survey population, but selection bias may limit the generalizability of our results. In addition, this is a heterogeneous population composed of individuals with different cancer types and treatment regimens; thus, not all adverse events may have been relevant to each individual. The race differences that we observed appear large, but because of the small number of black respondents, the results may not be reliable, so no definitive conclusions can be drawn. The study was also limited by the use of self-reporting. Since the respondents could have been treated up to 12 months before participating in the study, the responses may be subject to recall bias. Furthermore, since data were collected online and without confirmation by medical records or reports from physicians, it is possible that there were individuals who experienced an adverse event but either were unaware of it or did not understand the terminology used to describe it in the survey.

We were reassured to find that physician-patient discussions of adverse events were common, but it remains a concern that those discussions alone do not provide patients with sufficient understanding of the events, nor do they appear to adequately equip patients to cope with the adverse events that do occur. Efforts to improve cancer care should focus on developing tools to improve patients' understanding of the toxicities of chemotherapy, as well as providing resources to reduce the effects of adverse events.
